# Optimal Serum 25(OH)D Level and Vitamin D Intake in Young Korean Women

**DOI:** 10.3390/nu14224845

**Published:** 2022-11-16

**Authors:** Hye Ran Shin, Hyeon Ju Park, Sun Yung Ly

**Affiliations:** Department of Food and Nutrition, Chungnam National University, Daejeon 34134, Republic of Korea

**Keywords:** vitamin D, bone disease, young Korean women

## Abstract

Vitamin D status is essential for preventing bone disease. Young Korean women have the highest vitamin D deficiency prevalence compared with other demographic groups. This study aimed to establish the optimal vitamin D intake level for maintaining an adequate serum 25-hydroxyvitamin D (25[OH]D) level by season in young Korean women (mean age: 23.1 years). Each participant (wintertime, *n* = 101; summertime, *n* = 117) completed a lifestyle survey, dietary record, bone mineral density, and biochemical tests. Seasonal factors impacting 25(OH)D were identified, vitamin D intake for sufficient 25(OH)D levels was calculated, and the relationship between 25(OH)D and intact parathyroid hormone (iPTH) was analyzed. During summertime, 25(OH)D levels were higher than in wintertime (17.9 vs. 15.0 ng/mL). A 1 µg/1000 kcal increase in vitamin D intake increased 25(OH)D levels by 0.170 ng/mL in wintertime and 0.149 ng/mL in summertime. iPTH levels reached a theoretical plateau corresponding to an 18.4 ng/mL 25(OH)D level. The vitamin D intake threshold for maintaining 25(OH)D levels at ≥20 and ≥18.4 ng/mL was ≥10.97 μg/day. For a sufficient level of 25(OH)D in young Korean women, increasing summertime UV irradiation time and increasing vitamin D supplements and vitamin D-containing foods throughout the year is beneficial.

## 1. Introduction

Serum 25-hydroxyvitamin D (25[OH]D) levels are known to reduce the incidence of osteoporosis [1,2]. Osteoporosis is a major cause of poor quality of life in old age and fracture morbidity and mortality rates are very high [3]. Adequate dietary intake of calcium and vitamin D reduces the risk of fractures in older age [4] and high serum 25(OH)D levels decrease the risk of hip fractures [1]; thus, vitamin D supplementation has been reported to reduce the risk of falls [5]. As such, the nutritional status of vitamin D is important for prevention of bone diseases. However, vitamin D status among individuals is known to be poor worldwide [6], and the nutritional status of vitamin D among Koreans is critical [7].

Bone mineral density (BMD) increases during growth, particularly from puberty until the age of 25–35 years, when growth is complete [8]. At this time, peak bone mass is reached and subsequently, aging begins and bone mass is lost [9]. The degree of maximum bone mass and the rate of bone loss are affected by the interaction of genetic, hormonal, environmental, and nutritional factors [10]. In particular, the incidence of fractures and osteoporosis is very high in older women as their bone density decreases rapidly after menopause [11]. As women with higher peak bone mass may have a lower risk of fractures or osteoporosis after menopause [8], bone health in young women is important in relation to quality of life in old age.

Vitamin D also plays a critical role in women’s lives during pregnancy. Several studies have shown that vitamin D deficiency is a risk factor for pregnancy- and lactation-related osteoporosis, which can lead to increased musculoskeletal pain, decreased muscle strength, and often severe hip or back pain during pregnancy, which can lead to a decreased quality of life [12,13]. Furthermore, adequate vitamin D nutritional status during pregnancy improves gestational anemia and is an indicator of fetal nutrition [14]. Consequently, the vitamin D nutritional status of women in their twenties, who are preparing for pregnancy, is crucial.

On the other hand, when the vitamin D level in the blood is insufficient, calcium absorption is reduced. Decreased calcium levels induce parathyroid hormone (PTH) secretion to increase active vitamin D production, thereby increasing calcium absorption. As a result, it promotes bone turnover and causes bone loss. This phenomenon is called secondary hyperparathyroidism and vitamin D deficiency is considered to be the basic mechanism in the pathogenesis of osteoporotic fractures [15]. Many studies have been conducted based on this hypothesis to find the serum 25(OH)D level at which the PTH level is not increased [16,17,18]. 

The blood vitamin D level depends on both the amount of vitamin D synthesized under the skin through sunlight and on the intake of vitamin D from food or supplements. As vitamin D synthesis in the skin decreases during the seasons Which offer is less sunlight irradiation, vitamin D intake becomes important for maintenance of blood vitamin D concentration [19,20,21]. The Dietary Reference Intakes for Koreans 2020 (2020 KDRIs) sets the adequate intake of vitamin D for adults aged 19 to 64 years as 10 µg/day [22]. However, studies that have confirmed the relationship between bone health and vitamin D nutritional status in Korean subjects mainly focused on postmenopausal women and the elderly [23,24,25], which lacks the evidence criteria for setting adequate vitamin D intake levels for young adults. In 2009, 2009–2011, and 2013–2014 studies using the Korea National Health and Nutrition Examination Survey (KNHANES), it was reported that the vitamin D nutritional status of young Korean women had the highest deficiency prevalence compared with other age groups and men [7,19,26]. Blood tests for vitamin D have not been conducted in KNHANES since 2014; therefore, so studies to follow-up on the vitamin D status of young Korean women is urgently required.

Therefore, the purpose of this study was to identify the serum 25(OH)D levels required to maintain serum PTH levels and prevent bone loss. In addition, a dietary intake survey for the four seasons, a BMD, and blood and urine tests twice a year were analyzed to infer an optimal vitamin D intake for maintenance of an appropriate serum vitamin D level.

## 2. Materials and Methods

### 2.1. Study Participants

This study was conducted on women aged 19–29 years living in Daejeon in Korea. Participants were recruited using online community and offline campus bulletin boards. The selection criteria for participants in this study were those whose body mass index (BMI) was in the normal range (18 ≤ BMI < 23) and those who consumed regular meals at least twice a day. Patients with chronic, bone, and hormone-related diseases or other health disorders were excluded. Continued drug use, pregnancy, lactation, and mental illness were also exclusion criteria. The study was conducted from September 2020 to August 2021. Informed consent was obtained from all participants who were involved, prior to the start of the study.

The summertime (June–November) attendees were defined as individuals who underwent the biochemical test and completed both dietary intake surveys in autumn and summer; while the wintertime (December–May) attendees were defined as those who underwent the biochemical test and completed both dietary intake surveys in winter and spring. Participants who matched the selection criteria were screened and 140 individuals were recruited for each summertime and wintertime group; however, 24 and 37 participants dropped out from the summertime and wintertime group, respectively. Seventy individuals participated in both summertime and wintertime. Among the 118 summertime and 103 wintertime participants, 1 summertime and 2 wintertime participants with a daily vitamin D intake of ≥100 µg were excluded. Finally, a total of 218 young Korean women (117 summertime and 101 wintertime) were included. This study was performed in accordance with the Declaration of Helsinki and was approved by the Institutional Review Board of Chungnam National University (202005-BR-047-01).

### 2.2. General and Lifestyle Characteristics

The questionnaire on general and lifestyle characteristics was conducted by the self-reporting method once each in wintertime and summertime. The general questions included age and occupation (students, workers, other). Lifestyle characteristics, including ultraviolet (UV) exposure time, smoking status (no: not smoking for over 6 months), alcohol consumption (no: not drinking alcohol for over 6 months), physical activity, number of ways to avoid UV exposure, and vitamin D supplementation, were evaluated. For the UV exposure time, the total time spent in outdoor activities on one day during the week and one day on the weekend was self-recorded. Of these, the outdoor activity time from 10 a.m. to 3 p.m., the time when UV rays are strongest, was calculated in minutes (min), and the average value of UV exposure time for 2 days was used for analysis. Participants were asked to give multiple responses to ways in which they avoid UV exposure, such as sunscreen, cosmetics with UV-blocking effects, hats, facial masks, parasol, and other methods. Physical activity was investigated via the International Physical Activity Questionnaire short form Korean version [27]. Physical activity was classified into three categories by calculated metabolic equivalents of task (MET): low (<600 MET × min per week), moderate (600–3000 MET × min) and high (>3000 MET × min) physical activity. Vitamin D supplements consumed by participants were investigated, including supplement name, vitamin D content per dose, dose size, and daily intake frequency. The intake of vitamin D supplements per day was calculated as follows:

vitamin D content per serving × daily intake frequency

### 2.3. Dietary Assessment

For the dietary assessment, participants submitted their dietary records for 3 days, including 2 weekdays and 1 weekend day for each season (Spring: March–May; Summer: June–August; Autumn: September–November; Winter: December–February). Before start of the study, the participants were provided with a video guide on reporting dietary records, and all menu items, ingredients, and amounts consumed by them were recorded in a self-reported form. A ruler of the same length was provided to each participant, which was to be placed on the table while eating and a picture was to be taken. so that they could put it on the table while eating and take a picture. A trained investigator received all the photos, compared them with the participant’s self-reported record, confirmed the intake, and analyzed them. Food intake was analyzed using CAN-Pro 5.0 (web version) developed by the Korean Nutrition Society [28]. The vitamin D content of food was analyzed using the vitamin D database (DB) 2.0, published in 2022 [19]. When entering the dietary records of the participants, the food DB of CAN-Pro was used as a basis. However, if a food was not listed on the CAN-pro, the food DB entry was altered to be entered as closely as feasible to the meal composition of the participants, or a new food DB entry was established and utilized for analysis.

### 2.4. Anthropometry, Bone Turnover Markers (BMTs), and BMD

Anthropometry and biochemical tests were performed on request at the examination center located in Daejeon. Anthropometry was measured using an automatic height measuring device (X-SCAN PLUS Ⅱ, Jawon Medical Co., Ltd., Seoul, Republic of Korea). For the blood tests, 10 mL of venous blood was collected from the brachial vein on an empty stomach in the morning using a vacutainer serum separating tube (SST, BD Vacutainer, Netherlands). Blood was centrifuged at 3000 rpm for 10 min to separate serum and was stored in a −75 °C cryogenic freezer until analysis. An automated direct competitive chemiluminescent immunoassay (CLIA, ADVIA Centaur Vitamin D Total, Siemens, Washington DC, WA, USA) was used to determine the serum 25(OH)D level, which detects both 25-hydroxyvitamin D2 and D3. This method has been standardized for liquid chromatography with tandem mass spectrometry [29]. In this study, with reference to the value reported by the American Institute of Medicine and the Endocrine Society of the United States, serum 25(OH)D < 20 ng/mL was defined as deficiency [30,31]. Intact PTH (iPTH) was analyzed by electrochemiluminescence immunoassay (ECLIEA, Elecsyes PTH, Roche, Indianapolis, IN, USA). The reference value of iPTH for women is 15–65 pg/mL. Serum calcium was analyzed by colorimetric method with o-cresolphthalein complexone (oCPC, Clinimate CA, Sekisui Medical Co., Ltd., Tokyo, Japan). The reference value of serum calcium for women is 8.7–10.3 mg/dL. Bone alkaline phosphatase (BAP) was analyzed by chemiluminescence immunoassay (CLIA, Access Ostase Bone Specific Alkaline Phosphatase, Beckman Coulter Diagnostics, Brea, CA, USA). The reference value of BAP for premenopausal women is ≤14.0 µg/L. Urine was collected for N-Telopeptide type Ⅰ collagen (NTX) analysis at 10 mL each, stored in a conical tube at 4 °C, and measured by the CLIA method (VITROS Immunodiagnostic Products NTX Reagent Pack; Ortho-Clinical Diagnostics, Bridgend, Wales, UK). This value is presented in nanomoles of bone collagen equivalents per liter per millimole of creatinine per liter (nM BCE/mM Cr). The reference value of NTX for premenopausal women is 17–94 nM BCE/mM Cr. The posterior anterior lumbar spine (vertebrae L1–L4) was examined using a dual-energy X-ray absorptiometry instrument (ARIA BHR-1-76, GE Healthcare Korea Co., Ltd., Seongnam, Republic of Korea) to measure areal BMD (g/cm^2^).

### 2.5. Statistical Analysis

The results of the study were obtained from the analysis of winter or summertime survey attendees and the total participants who were combined with summertime and wintertime study attendees. For general and lifestyle characteristics, seasonal nutrient intake was calculated by mean ± standard error of mean for continuous variables and proportions were calculated for categorical variables. The scatter plot and mean were calculated for the results of seasonal serum and urine analysis (serum 25[OH]D, iPTH, BAP, Calcium, urine NTX, and BMD). To verify the difference between wintertime and summertime results, the Student’s *t*-test for independent samples was used. The seasonal influencing factors of serum 25(OH)D levels were verified using a stepwise method of multiple linear regression analysis. As independent variables, age, BMI, 10 a.m. to 3 p.m. outdoor time (UV exposure time), occupation, smoking, alcohol consumption, number of ways to avoid UV exposure, physical activity, and vitamin D intake (per 1000 kcal) were used. A stepwise regression was performed, and a *p* value > 0.10 was used for variable removal.

The relationship between 25(OH)D and iPTH was analyzed using an exponential decay model for total participants. Serum 25(OH)D levels required to achieve plateau iPTH concentrations were calculated using the following method [32,33]:

iPTH = a + b × exp (c × 25[OH]D)

The receiver operating characteristic (ROC) analysis and Youden’s index calculation were used to calculate the optimal vitamin D consumption to maintain serum 25(OH)D sufficiency for the whole population. Area under the ROC curve (AUC) and *p*-values were provided. Pearson’s correlation coefficient was used to examine the relationship between serum 25(OH)D levels, BMTs (iPTH, NTX, BAP, and calcium), and BMD.

SPSS software, version 27.0 (IBM Corp., Armonk, NY, USA), was used for statistical analysis and MedCalc^®^ Statistical Software, version 20.111 (MedCalc Software Ltd., Ostend, Belgium), was used to compare the AUCs. Exponential decay was analyzed through GraphPad Prism 9, Version 9.4.0 (GraphPad Software, Inc., San Diego, CA, USA). A *p*-value < 0.05 was considered statistically significant and all tests were two-sided.

## 3. Results

### 3.1. The Status of Participants

Table 1 presents the general and lifestyle characteristics of all the participants. The average age of participants was 23.1 ± 0.16 years and there was no significant difference between wintertime and summertime attendees. There was no significant difference in height, weight, and BMI between the two groups, and the UV exposure time between 10 a.m. and 3 p.m. was 19.5 min in summertime and 14.5 min in wintertime (*p* = 0.052). There was no significant difference in the distribution between the two groups in occupation, smoking status, alcohol consumption status, number of ways to avoid UV exposure, and physical activity level, however, a greater number of people took vitamin D supplements in the summertime group (*p* < 0.05). The proportion of participants with a serum 25(OH)D level of less than 20 ng/mL was 82.2% (83 attendees) in wintertime and 65.8% (77 attendees) in summertime.

### 3.2. Serum Levels of 25(OH)D, Calcium, BMTs, and BMD

**Figure 1:**
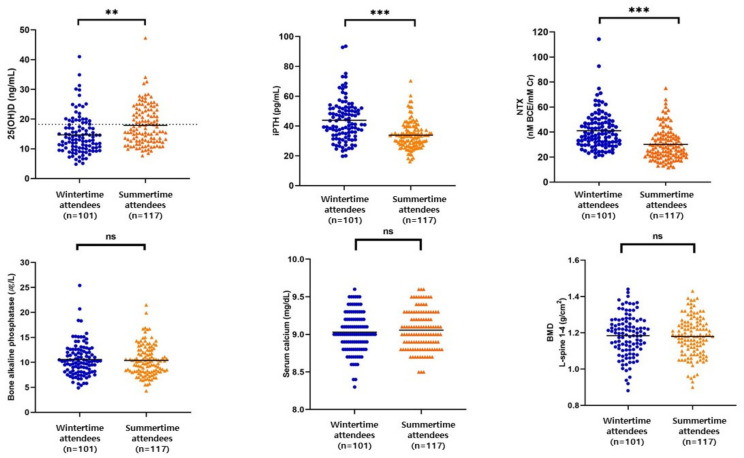
Scatter plots of bone turnover markers and bone mineral density. Solid line: mean value, dotted line on the 25(OH)D graph: 20 ng/mL; iPTH, intact parathyroid hormone; NTX, type Ⅰ collagen cross-linked N-telopeptide; BMD, bone mineral density; *** *p* < 0.001, ** *p* < 0.01, ns: not significant.

Figure 1 shows the results of the bone biomarkers of study participants and the difference between the two seasons. The mean serum 25(OH)D level was significantly higher (*p* < 0.01) in summertime (17.9 ± 0.61 ng/mL) than in wintertime (15.0 ± 0.64 ng/mL). iPTH levels was significantly higher in wintertime (43.9 ± 1.40 pg/mL) than in summertime (33.78 ± 0.84 pg/mL, *p* < 0.001). NTX levels were significantly higher (*p* < 0.001) in wintertime (41.0 ± 1.51 nM BCE/mM Cr) than in summertime (30.18 ± 1.16 nM BCE/mM Cr). However, BAP (10.52 ± 0.32 µg/L for wintertime, 10.68 ± 0.37 µg/L for summertime), serum calcium (9.02 ± 0.02 mg/dL for wintertime, 9.05 ± 0.02 mg/dL for summertime), and BMD (1.18 ± 0.11 g/cm^2^ for wintertime, 1.17 ± 0.01 g/cm^2^ for summertime) did not differ significantly between the two seasons.

### 3.3. Seasonal Nutrient Intake

There were no significant differences in total energy intake, calcium intake, vitamin D intake from foods, and vitamin D intake through supplements by season (Table 2). In this study, dietary intake of vitamin D was determined according to a previous research which showed that the contribution of food groups from fish and shellfish, eggs, meat and meat products, milk and dairy products, mushrooms, and grains and grain products to vitamin D intake in Koreans is 99% [19]. In both seasons, dietary intake of vitamin D from fish and shellfish was the highest (contribution to vitamin D intake was 64.9% in wintertime and 54.4% in summertime); however, there was no significant difference in vitamin D intake from all major food groups between the two seasons groups.

### 3.4. Multiple Linear Regression of Factors Affecting Serum 25(OH)D Level by Season

As a result of multiple linear regression analysis of factors affecting serum 25(OH)D levels in the 218 total participants, the influencing factors of vitamin D intake including supplements per 1000 kcal, ways to avoid UV exposure, and UV exposure time were chosen as variables (Table 3). Serum 25(OH)D levels increased by 0.170 ng/mL for every 1 µg/1000 kcal increase in vitamin D intake (*p* < 0.001). The serum 25(OH)D levels decreased by 1.577 ng/mL for each increase in the participants’ number of ways to avoid UV exposure (*p* < 0.001), whereas the serum 25(OH)D levels increased by 0.056 ng/mL for every 1 min increase in UV exposure time (*p* < 0.05). In wintertime, vitamin D intake was the only factor affecting serum 25(OH)D level and in summertime, vitamin D intake, number of ways to avoid UV exposure, and UV exposure time were analyzed as influencing factors. When vitamin D intake was increased by 1 µg/1000 kcal, serum 25(OH)D levels increased by 0.170 ng/mL and 0.147 ng/mL in wintertime and summertime, respectively (*p* < 0.05). When the number of ways to avoid UV exposure in summertime increased, the serum 25(OH)D level decreased by 2.559 ng/mL (*p* < 0.001) and when the UV exposure time was increased by 1 min, the serum 25(OH)D level increased by 0.059 ng/mL (*p* < 0.05).

### 3.5. Exponential Decay for Optimal Serum 25(OH)D to Suppress PTH Elevation

**Figure 2:**
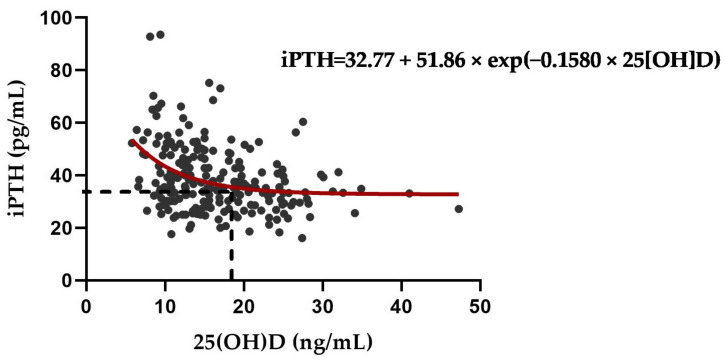
Nonlinear regression between serum 25(OH)D and intact PTH levels in total participants. Serum 25(OH)D levels required to achieve plateau iPTH concentrations were calculated using the following equation: iPTH = a + b × exp (c × 25[OH]D). iPTH increased when the 25(OH)D concentration decreased to 18.44 ng/mL or less.

As a result of calculating the changes in 25(OH)D and iPTH using an exponential decay function, iPTH increased when the 25(OH)D concentration decreased to 18.44 ng/mL or less (Figure 2). Our resulting equation was as follows:

iPTH = 32.77 + 51.86 × exp (−0.1580 × 25[OH]D)

### 3.6. ROC Analysis for Optimal Vitamin D Intake at Serum 25(OH)D ≥20 and ≥18.44 ng/mL

**Figure 3:**
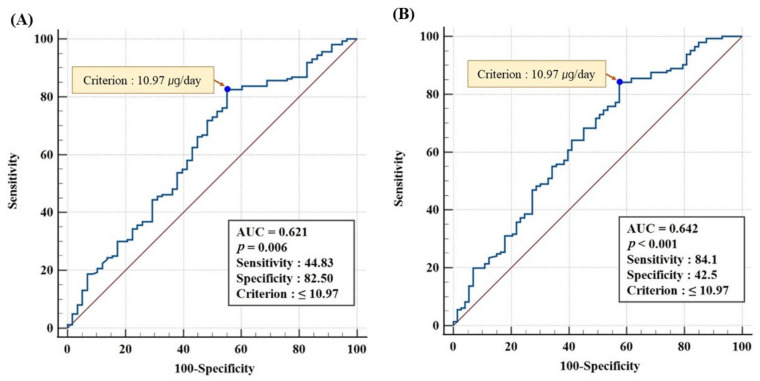
ROC analysis for assessing vitamin D intake at serum 25(OH)D levels ≥ 20 ng/mL (**A**) and ≥18.44 ng/mL (**B**). The vitamin D deficiency cutoff value, as per the American Institute of Medicine and the Endocrine Society of the United States, is 20 ng/mL (**A**) and the appropriate serum 25(OH)D level obtained from the total participants of this study was 18.44 ng/mL (**B**).

The ROC analysis was performed to determine the threshold of vitamin D intake to maintain serum 25(OH)D levels above 20 ng/mL (Figure 3). In this case, the optimal vitamin D intake criterion was 10.97 µg/day (AUC = 0.621, *p* < 0.01). Additionally, the threshold for vitamin D intake based on the serum 25(OH)D level of 18.44 ng/mL analyzed in this study was 10.97 μg/day (AUC = 0.642, *p* < 0.001).

### 3.7. Correlation between Variables

Serum 25(OH)D levels in both, total participants and the wintertime attendee cohort correlated negatively with iPTH (*p* < 0.001), but positively with serum calcium levels in summertime (*p* < 0.05, Table 4). In all cases, NTX and BAP were positively correlated (*p* < 0.001) and serum calcium levels in total participants and wintertime attendees were negatively correlated with iPTH (*p* < 0.05). However, serum 25(OH)D levels and all BMTs were not associated with BMD.

## 4. Discussion

Worldwide, vitamin D deficiency is a serious concern for all ages. Poor nutritional status of vitamin D in young women is a risk factor for post-menopausal fractures and osteoporosis [34]. Based on KNHANES, the serum 25(OH)D levels was found to be the lowest in women aged 19 to 29 years among all age groups; values were at 4.71 ng/mL in 2009 [26], 13.73 ng/mL in 2009–2011 [7], and 13.85 ng/mL in 2013–2014 [19]. In addition, their intake of vitamin D was 2.48 µg in 2009 [26], 2.77 µg in 2009–2011 [7], and 2.48 µg in 2013–2014 [19], which accounted for only 25% of the 2020 KDRIs vitamin D adequate intake value of 10 µg. Even though vitamin D deficiency is a national problem, blood vitamin D has not been measured in KNHANES since 2014. Therefore, this study was carried out to investigate the factors influencing vitamin D nutritional status in young Korean women aged 19–29 years in a metropolitan area, and to determine the vitamin D intake level required to maintain an optimal level of serum 25(OH)D.

The main contributing factor to blood vitamin D levels is the vitamin D synthesis under the skin caused by UV irradiation. In this study, the time spent outdoors during the daytime from 10 a.m. to 3 p.m. was found to be about 17.2 ± 1.28 min in young Korean women. The serum 25(OH)D level increased by 0.056 ng/mL when the UV exposure time was increased by 1 min in this study. A study conducted by Asakura et al. [20] in Japan showed that increasing UV exposure time in summer by 10 min increased serum 25(OH)D_3_ level by 0.47 ng/mL. Another study conducted by Goswami et al. [35] on Indian men in the summertime (August to September) found that serum 25(OH)D level increased by 2.03 ng/mL per hour of sun exposure, showing similar results to this study. 

In addition, serum 25(OH)D levels decreased by 1.57 ng/mL as the number of ways to avoid UV exposure increased. However, none of the variables associated with UV exposure in wintertime had a statistically significant effect on serum vitamin D levels. Under the skin, 7-dihydrocholesterol is synthesized effectively on exposure to UVB radiation in the 290–315 nm wavelength range. Korea lies between 33° and 38° north latitude and winter UVB radiation is only a quarter of summer levels [36]. Park et al. [37], who conducted a study on the UV exposure time required for vitamin D synthesis in Koreans, stated that the threshold exposure time for vitamin D synthesis in summer was less than 30 min, but more than 100 min in winter. It was also reported that sufficient vitamin D synthesis was impossible with UV exposure alone in winter. In particular, in the northern hemisphere, winter temperatures are low; therefore, people wear long sleeves and pants. As a result, skin is less exposed to UV radiation during outdoor activities, which also contributes to vitamin D deficiency.

Aside from UV exposure time, daily vitamin D intake, including supplement intake, was discovered to be an influential variable in this study. In total participants, it was found that the vitamin D intake increased by 1 µg/1000 kcal with serum vitamin D level increase of 0.170 ng/mL These effects of vitamin D intake were also shown in wintertime study. In a study conducted on the Japanese population, the effect of vitamin D intake on serum vitamin D level was reported to be higher than that observed in our study; an increase in vitamin D intake of 1 µg/1000 kcal increased serum 25(OH)D_3_ by 0.88 ng/mL in summer and 1.7 ng/mL in winter [20]. However, in a study that confirmed the serum 25(OH)D level as a result of vitamin D supplementation, when vitamin D was additionally consumed at 1000 IU/day (25 µg/day), serum 25(OH)D increased by 12 nmol/L (4.81 ng/mL) [38]. Another meta-analysis of the relationship between vitamin D-fortified foods and serum 25(OH)D levels revealed that intake of 100 IU/day (2.5 µg/day) dietary vitamin D could increase serum 25(OH)D by 2 nmol/L (0.80 ng/mL) [39]. Researchers report the effects of vitamin D intake on blood vitamin D levels slightly differently. In the latter two studies, the effects of vitamin D intake on serum vitamin D (0.19 ng/mL and 0.32 ng/mL of serum 25(OH)D per µg of vitamin D intake, respectively) were similar to or slightly higher than the results of this study. Previous research on Koreans has shown that vitamin D intake has a positive effect on serum 25(OH)D levels [7,26]. A study conducted on Koreans that confirmed a positive correlation between vitamin D intake and serum 25(OH)D level during low UV irradiation, pointed out the importance of vitamin D intake in winter [19]. In this study, the effect of vitamin D intake on serum 25(OH)D levels was greater in wintertime, thereby, supporting the results of previous studies.

In the KNHANES, serum vitamin D levels were measured only between 2008–2014. The average serum 25(OH)D concentration of Korean women aged 19–29 years in the 2013–2014 KNHANES was 13.85 ng/mL [19], which was lower than the 16.54 ng/mL for the total participants in this study. Expert groups, such as the American Institute of Medicine and the Endocrine Society of the United States, recommend maintaining serum 25(OH)D levels at 20 ng/mL [30,31]; however, research about young women in Korea has been limited. In this study, the serum 25(OH)D level, which showed a sharp increase in blood iPTH, was 18.44 ng/mL or less. This was similar to or slightly higher than the 46.9 nmol/L (18.79 ng/mL) [16] for Croatian women and the 44 nmol/L (17.63 ng/mL) [17] for African American women. As renal function declines with age, higher 25(OH)D levels are required in old age to prevent serum PTH elevation [17,40]. Therefore, it is considered desirable to maintain the serum vitamin D levels of young Korean women slightly above the results of this study.

In order to maintain the optimal serum 25(OH)D level of 18.44 ng/mL based on the iPTH level in this study, at least 10.97 µg/day of vitamin D should be ingested. This was the same even based on the vitamin D deficiency threshold of 20 ng/mL suggested by the American Institute of Medicine [30] and the Endocrine Society of the USA [31]. The ROC curve showed that the 18.44 ng/mL serum 25(OH)D level was closer to the upper left and had a greater AUC; hence, it was a stronger predictor of vitamin D intake than the 20 ng/mL serum 25(OH)D level. The optimal intake level for summertime attendees was found to be 10.97 µg/day, but no significant results were obtained for wintertime attendees (data not shown). As most participants’ serum 25(OH)D levels were very low in the wintertime, the ROC analysis had little meaning. In a study conducted on young Japanese women, it was found that the optimal vitamin D intake for maintaining a serum 25(OH)D level of 20 ng/mL was 11.6 µg/day, which was slightly higher than the level found in this study [41]. The survey period of this Japanese study was December-January and data from summer with higher UV irradiation were not included. As Japan is located in the southern part of the Northern Hemisphere than of Korea, the vitamin D requirement may have been lower if data from the summer survey were included. According to the 2020 KDRIs, the adequate intake of vitamin D for young adult women was set at 10 µg/day. As results (10.97 µg/day) for the optimal vitamin D intake of young Korean women derived from our study depended on the summer survey data, it is thought that adequate annual intake of vitamin D should be higher, considering vitamin D intake in winter when sunlight is scarce.

In order to reduce the risk of age-related BMD loss, it is crucial to begin consumption of sufficient calcium and vitamin D at a young age [42,43]. Although the average intake of vitamin D in total participants of our study was only 3.14 µg/day, the average vitamin D intake from food and supplements was 21.1 µg/day for the 55 participants, which was more than 2-folds higher than the 2020 KDRIs of vitamin D (10 µg/day). A previous study that analyzed the KNHANES found that vitamin D intake in Korean women aged 19–29 years was 2.48 µg/day, which was lower than the vitamin D intake of the participants of this study [19]. The daily calcium intake of the subjects in this study was 554.6 mg, which was higher than the calcium intake of 439.14 mg/day in Korean women aged 19–29 years, according to the 2020 KNHANES [42], but far below the 2020 KDRIs (700 mg/day) [22]. In a study on 2011 KNHANES, the average lumbar BMD of 19–29-year-old women was 0.97 ± 0.01 g/cm^2^, which was slightly higher than that of the subjects in this study [44]. In a study on the effect of combined calcium and vitamin D supplementation on BMD, it was reported that the increase in BMD was significantly higher than that of the control group when calcium and vitamin D were supplemented together in healthy young women aged 16–36 years [45]. In a study on 2009 KNHANES, the effect of vitamin D intake on BMD was better in the low calcium intake group [23]. The subjects in the previous study were Korean women over the age of 50. Several other studies have also shown that vitamin D intake becomes more crucial in groups with low calcium intake [46,47]. Given that young Korean women consume more dairy products than older women, their calcium intake can be increased easily, but not dietary vitamin D intake [48]. Vitamin D fortification in food is not yet mandatory in Korea; therefore, it must be implemented soon.

Higher NTX and iPTH concentrations in young Korean women in wintertime than in summertime suggest that bone remodeling occurs more in wintertime. Previous studies have reported that BTMs increase in winter when vitamin D nutritional status is poor [49,50]. As this study included young females in whom bone remodeling is continuously observed, the relationship with BMD may not have been observed. In this study, there was no statistically significant correlation between BTMs and BMD as in some previous studies on young adult women [51,52]. BTMs provide a measure of overall bone turnover, while BMD measures specific areas of the skeletal system. Elevated BTMs are associated with increased bone turnover, which cause deteriorated bone quality and increased risk of fracture [53]. BTMs are negatively correlated with BMD, which is known to increase with age [54,55].

This study is the first to determine the optimal serum 25(OH)D level for women in their twenties in Korea and the optimal vitamin D intake level in Korea. This study had the additional strength of identifying seasonal factors that influence serum 25(OH)D levels. However, there were some limitations to this study. First of all, as this study was conducted on a relatively small number of women living in a major Korean city, there may have been a problem with the representativeness of the sample. Approximately 70% of the study participants were college students, came from various provinces of Korea, and had a slightly higher economic level than that the average of young women in Korea. Secondly, due to the COVID-19 pandemic, a reduction in time spent outdoors may have affected the serum 25(OH)D level in the study participants. Therefore, the situation might have differed slightly from that at the time of a normal life pattern. Thirdly, because BMD was only measured at the lumbar spine, correlations with BMD measured at other locations could not be confirmed. However, one study showed that bone density in different regions are correlated with each other, and the correlation is greater with younger age [56].

## 5. Conclusions

It was confirmed that the serum 25(OH)D levels for the prevention of bone loss is 18.44 ng/mL or higher and vitamin D intake should be higher than 10.97 μg/day to prevent vitamin D deficiency in young Korean women. It seems reasonable to use the current level of 25(OH)D > 20 ng/mL as the KDRI for vitamin D as a target for bone health in young Korean women. However, as the current intake of vitamin D in young Korean women was very low, the intake of vitamin D must be increased by at least three times the current levels. To do this, the Korean government should implement a mandatory fortification for vitamin D in food, which should be argued prior to vitamin D supplementation to improve Korea’s national nutrition status. In countries with vitamin D food fortification policies, vitamin D fortification is approximately 22–200 IU per serving of food [57]. Based on this standard, if Korea sets the vitamin D fortification standard to 5 μg/serving or less in foods such as grains, dairy products, and fruit juice, even making allowance for supplement intake or duplicate intake of food, in is unlikely that the standard upper limit of 100 μg/day vitamin D intake for adults aged 19 years or older would be exceeded.

## Figures and Tables

**Table 1:** General and lifestyle characteristics of the participants.

		Wintertime Attendees	Summertime Attendees	Total Participants	*p*-Value
		(*n* = 101)	(*n* = 117)	(*n* = 218)	
Age (years)		23.2 ± 0.25	23.02 ± 0.22	23.1 ± 0.16	0.527
Height (cm)		162.0 ± 0.56	162.3 ± 0.51	162.1 ± 0.34	0.753
Weight (kg)		56.2 ± 0.96	56.2 ± 0.85	56.2 ± 0.63	0.921
BMI (kg/m^2^)		21.5 ± 0.28	21.5 ± 0.25	21.5 ± 0.18	0.853
UV exposure time (min)		14.5 ± 1.82	19.5 ± 1.77	17.2 ± 1.28	0.052
Occupation	Student	70 (69.3)	84 (71.8)	154 (70.6)	0.921
	Worker	19 (18.8)	20 (17.1)	39 (17.9)	
	Other	12 (11.9)	13 (11.1)	25 (11.5)	
Smoking status	Yes	7 (6.9)	9 (7.7)	16 (7.3)	0.830
	No	94 (93.1)	108 (92.3)	202 (92.7)	
Alcohol consumption	Yes	76 (75.2)	88 (75.2)	164 (75.2)	0.995
	No	25 (24.8)	29 (24.8)	54 (24.8)	
Number of ways to avoid UV exposure	Never	11 (10.8)	6 (5.1)	17 (7.8)	0.414
	1	24 (23.8)	29 (24.8)	53 (24.3)	
	2	33 (32.7)	37 (31.6)	70 (32.1)	
	≥3	33 (32.7)	45 (38.5)	78 (35.8)	
Physical activity	Low	40 (39.6)	57 (48.7)	97 (44.5)	0.401
	Moderate	41 (40.6)	40 (34.2)	81 (37.2)	
	High	20 (19.8)	20 (17.1)	40 (18.3)	
Taking vitamin D supplements	Yes	19 (18.8)	36 (30.8)	55 (25.2)	0.043
	No	82 (81.2)	81 (69.2)	163 (74.8)	
Prevalence of 25(OH)D deficiency	Deficiency (<20 ng/mL)	83 (82.2)	77 (65.8)	160 (73.4)	0.006
	Sufficiency (≥20 ng/mL)	18 (17.8)	40 (34.2)	58 (26.6)	

Mean ± SEM or N(%); UV; Ultraviolet; UV exposure time: UV rays were strongest between 10 a.m. and 3 p.m.; Students: college students or higher; worker: full-time worker; other: unemployed or part-time workers; Physical activity: Low, <600 metabolic equivalents of task [MET] × min per week; moderate, 600–3000 MET × min per week; High, >3000 MET × min.

**Table 2:** Energy, calcium, and vitamin D intake in wintertime and summertime.

	Wintertime Attendees	Summertime Attendees	Total Participants	*p*-Value
Dietary intake of nutrients *(n* = 218)				
Energy (kcal/day)	1390.5 ± 37.0	1458.8 ± 36.9	1427 ± 26.2	0.195
Calcium (mg/day)	533.8 ± 23.0	572.46 ± 21.4	554.55 ± 15.7	0.220
Vitamin D (µg/day)	3.19 ± 0.36	3.09 ± 0.29	3.14 ± 0.22	0.827
Dietary intake of vitamin D rich food groups and contribution (µg/day, % ^§^)				
Fish and shellfish	2.07 ± 0.35 (64.9)	1.68 ± 0.28 (54.4)	1.86 ± 0.22 (59.2)	0.393
Eggs	0.64 ± 0.06 (20.1)	0.73 ± 0.04 (23.6)	0.69 ± 0.04 (22.0)	0.248
Meat and meat products	0.24 ± 0.01 (7.5)	0.33 ± 0.06 (10.7)	0.29 ± 0.04 (9.2)	0.196
Milk and dairy products	0.12 ± 0.03 (3.8)	0.11 ± 0.02 (3.6)	0.12 ± 0.02 (3.8)	0.913
Mushrooms	0.08 ± 0.01 (2.5)	0.10 ± 0.01 (3.2)	0.09 ± 0.01 (2.9)	0.321
Grain and grain products	0.04 ± 0.02 (1.3)	0.06 ± 0.02 (1.9)	0.05 ± 0.02 (1.6)	0.602
Total vitamin D intake (µg/day)				
With supplements ^†^ (*n* = 55)	24.7 ± 3.22	19.2 ± 2.70	21.1 ± 2.10	0.218
Without supplements ^‡^ (*n* = 163)	3.32 ± 0.41	3.17 ± 0.36	3.25 ± 0.27	0.789
Overall (*n* = 218)	7.34 ± 1.07	8.10 ± 1.10	7.75 ± 0.77	0.998

Mean ± SEM; ^§^ Percentage of total dietary vitamin D intake contributions; ^†^ Taking vitamin D supplements: participants taking vitamin D supplements (wintertime, *n* = 19; summertime, *n* = 36); ^‡^ Not taking vitamin D supplements: participants not taking vitamin D supplements (wintertime, *n* = 82; summertime, *n* = 81).

**Table 3:** Regression coefficients for the association between serum 25(OH)D levels and variables by season.

Season	Variable	Regression Coefficient	95% CI	*p* Value
Total participants	Vitamin D intake (with supplement, µg/1000 kcal)	0.170	0.078, 0.263	<0.001
	Way to avoid UV exposure	−1.577	−2.453, −0.702	<0.001
	UV exposure time (min)	0.056	0.012, 0.101	0.013
Wintertime attendees	Vitamin D intake (with supplement, µg/1000 kcal)	0.170	0.030,0.310	0.018
Summertime attendees	Way to avoid UV exposure	−2.559	−3.758, −1.360	<0.001
	Vitamin D intake (with supplement, µg/1000 kcal)	0.147	0.028, 0.266	0.016
	UV exposure time (min)	0.059	0.003, 0.115	0.039

UV exposure time: UV rays were strongest between 10 a.m. and 3 p.m.

**Table 4:** Correlation between serum 25(OH)D levels and bone turnover marker levels.

Season	Variables	iPTH	NTX	BAP	Serum Calcium	BMD (L1-4)
Total participants	25(OH)D	−0.302 ***	−0.095	−0.070	0.092	−0.045
	iPTH		0.132	−0.056	−0.134 *	0.053
	NTX			0.480 ***	0.025	−0.087
	BAP				0.037	−0.133
	Serum calcium					−0.010
Wintertime attendees	25(OH)D	−0.335 ***	−0.072	−0.109	−0.037	0.011
	iPTH		−0.058	−0.074	−0.202 *	0.068
	NTX			0.589 ***	−0.063	−0.151
	BAP				−0.083	−0.187
	Serum calcium					0.013
Summertime attendees	25(OH)D	−0.134	0.036	−0.056	0.183 *	−0.086
	iPTH		0.052	−0.033	−0.018	0.015
	NTX			0.490 ***	0.169	−0.053
	BAP				0.116	−0.094
	Serum calcium					−0.029

Pearson’s correlation coefficient. iPTH, intact parathyroid hormone; NTX, type Ⅰ collagen cross-linked N-telopeptide; BMD, bone mineral density; L1–4, lumbar spine 1–4; *** *p* < 0.001, * *p* < 0.05.

## Data Availability

The data presented in this study are available on request from the corresponding author.
